# Endothelial progenitor cells promote osteogenic differentiation in co-cultured with mesenchymal stem cells via the MAPK-dependent pathway

**DOI:** 10.1186/s13287-020-02056-0

**Published:** 2020-12-11

**Authors:** Chu Xu, Haijie Liu, Yuanjia He, Yuanqing Li, Xiaoning He

**Affiliations:** 1 Department of Stomatology, The 4th Affiliated Hospital of China Medical University, No.4 Chongshan Dong Road, Shenyang, 110032 Liaoning China; 2 Department of General Dentistry, School of Stomatology, China Medical University, Shenyang, 110001 Liaoning China

**Keywords:** Endothelial progenitor cells, Mesenchymal stem cells, Osteogenesis, MAP kinase signaling pathway, Co-culture

## Abstract

**Background:**

The role of bone tissue engineering is to regenerate tissue using biomaterials and stem cell-based approaches. Combination of two or more cell types is one of the strategies to promote bone formation. Endothelial progenitor cells (EPCs) may enhance the osteogenic properties of mesenchymal stem cells (MSCs) and promote bone healing; this study aimed to investigate the possible mechanisms of EPCs on promoting osteogenic differentiation of MSCs.

**Methods:**

MSCs and EPCs were isolated and co-cultured in Transwell chambers, the effects of EPCs on the regulation of MSC biological properties were investigated. Real-time PCR array, and western blotting were performed to explore possible signaling pathways involved in osteogenesis. The expression of osteogenesis markers and calcium nodule formation was quantified by qRT-PCR, western blotting, and Alizarin Red staining.

**Results:**

Results showed that MSCs exhibited greater alkaline phosphatase (ALP) activity and increased calcium mineral deposition significantly when co-cultured with EPCs. The mitogen-activated protein kinase (MAPK) signaling pathway was involved in this process. p38 gene expression and p38 protein phosphorylation levels showed significant upregulation in co-cultured MSCs. Silencing expression of p38 in co-cultured MSCs reduced osteogenic gene expression, protein synthesis, ALP activity, and calcium nodule formation.

**Conclusions:**

These data suggest paracrine signaling from EPCs influences the biological function and promotes MSCs osteogenic differentiation. Activation of the p38MAPK pathway may be the key to enhancing MSCs osteogenic differentiation via indirect interactions with EPCs.

## Background

Bone defects are a major clinical issue in terms of functional reconstruction and remodeling appearance. Although autografts are still considered to be the best material for tissue replacement, it still has concerns including the additional surgical procedure, donor site mobility, and quantity limitations. Bone tissue engineering, based on stem cells, is a promising strategy in regenerating bone defects [1, 2]. The basic concept in bone tissue engineering is to generate new bone by combining osteo-potential cells and growth factors, in combination with suitable scaffolds, and specific osteogenic cells or their progenitors [3–5].

However, bone tissues are sophisticated structures formed by several cell types and their surrounding extracellular matrix. Extracellular matrix and cell organization are essential to bone development and repairment, which are highly associated with the functions of tissue. Cells interact with the extracellular matrix and the cells surrounded the tissues to maintain the biological structure and function of the tissue [6]. Thus, it is important to understand relationships between each cell type to reach successful bone regeneration. For these reasons, researchers have turned to co-culture investigations to enhance the regeneration of bone [7]. Co-culture is a strategy to culture multiple cell types in the same environment and controls the behavior of cells through the direct or indirect interaction between cells to drive tissue formation. Vascularization is a crucial step in bone development, and correct vascularization distributes oxygen and nutrients to tissue, removes waste products, and promotes bone formation [8, 9]. So, bone equivalents should contain both osteoprogenitor and vascular cells, which enhance and aid the development of bone constructs and enabling survival as well as bone integration in the defect areas.

Mesenchymal stem cells (MSCs) are important sources of cell therapy in tissue engineering [10]. They are isolated from different sources and differentiated into several lineages, e.g., osteogenic and chondrogenic, under suitable induction conditions [11]. MSCs induce direct effects through cellular signaling via physical contacts and secrete chemokines and cytokines for paracrine and autocrine functions, to generate trophic support in tissue regeneration [12, 13].

Endothelial progenitor cells (EPCs) have the capacity to differentiate into mature endothelial cells, and the role of EPCs in angiogenesis has been studied extensively, both in vitro and in vivo [14, 15]*.* EPCs are able to promote the regeneration of bone in host bone defects, which might be the consequence of an increased vessel formation and other cellular/molecular factors involved in bone healing [16]. Because of the outstanding ability, these cells are widely used in tissue engineering approaches. Hence, both EPCs and MSCs are promising candidates for bone tissue engineering and large segmental bone defect therapies [17].

The occurrence of EPCs with MSCs has profound effects on cell proliferation and differentiation [18, 19]. Investigations have shown that co-implantation of EPCs and MSCs significantly improves bone formation [20–22], but the relationship between MSCs and EPCs in osteogenesis is not fully elucidated. The cellular crosstalk between EPCs and MSCs involves paracrine mechanisms based on several cytokine factors, and direct cell-to-cell communications via gap junctions might promote MSC osteogenic differentiation [16, 23]. An understanding of the cellular and molecular interactions of EPCs and MSCs will enhance the successful development of bone regeneration.

Therefore, we used MSCs and EPCs to study their osteogenic interaction in co-culture experiments, to identify gene expression changes driving this interaction, to determine how EPCs influence osteogenic differentiation of MSCs, and to investigate the involvement of mitogen-activated protein kinase (MAPKs) pathways in the osteogenic differentiation.

## Material and methods

### Isolation of MSCs

Investigations were conducted in accordance with institutional ethical standards, the Declaration of Helsinki, and national and international guidelines. The study was approved by the China Medical University Animal Care and Use Committee. Sprague-Dawley (SD) rats, 4–6 weeks old, were euthanized by CO_2_. Rat femurs and tibias were dissected in a sterile hood, and bone marrow cells were collected in sterile phosphate-buffered saline (PBS). BMSCs were separated by Histopaque-1083 (1.077 g/ml, Sigma-Aldrich, USA) density gradient centrifugation at 400*g* for 20 min and placed in a flask at a density of 1 × 10^6^ cells/ml. Cells were cultured at 37 °C and 5% CO_2_. The cells will be passaged using 1x TrypLE express (Invitrogen, USA) when the confluency reached 70–80% after 5–7 days.

### Isolation of EPCs

EPC preparation and culture were performed as previously described [24]. Briefly, bone marrow mononuclear cells were washed twice in PBS and suspended in EPC media (EGM-2 media supplemented with growth factor bullet kit (Lonza, Germany), at a density of 1 × 10^6^ cells/ml). After 24 h incubation at 37 °C and 5% CO_2_, non-adherent cells were removed and fresh medium was added. After 5–7 days, 90% confluence adhered cells were split for passage.

### Flow cytometry analysis and immunofluorescence staining

MSCs and EPCs were harvested at passage 4 for flow cytometry analysis and immunofluorescence staining. Briefly, 1 × 10^6^ cells were washed with 10% FBS/PBS and centrifuged at 1000 rpm, 5 min to gather a pellet for flow cytometry analysis; MSCs were stained with FITC-conjugated rat anti-CD44, FITC-conjugated rat anti-CD90, FITC-conjugated rat anti-CD31, and FITC-conjugated rat anti-CD34 antibodies at a concentration of 2 mg/ml at 4 °C. EPCs were stained with rat anti-CD31, rat anti-CD34, FITC-conjugated rat anti-CD11b, and FITC-conjugated rat anti-CD133; mouse IgG was served as negative controls. Cells were examined by flow cytometry with 10,000 events recorded for each condition. The results were analyzed by Flowjo software. For immunofluorescence staining, EPCs were co-stained with DPBS-E containing 10 μg/ml Dil-labeled acLDL and FITC-conjugated lectin (QiYue Technologies, China) for 1 h at 37 °C. Preparations were then observed under fluorescence microscopy.

### Experimental groups and induction culture conditions

Co-culture of MSCs and EPCs were established by using Transwells (Corning, USA). EPCs were incubated in the upper chamber, and MSCs were inoculated in the lower chamber at a density of 5 × 10^5^ cells/cm^2^for both cell types (co-MSCs). Monolayer culture MSCs in a 6-well plate at a density of 5 × 10^5^ cells/cm^2^ were used as control (MSCs). The cell culture media was osteogenic inducing media, with a final concentration of 10 nM dexamethansone, 50 μg/ml ascorbic acid, and 10 mM β-Glycerophosphate. Cells were incubated for 48 h at 37 °C at 5% CO_2_. For specific inhibitors treatments, co-cultured MSCs were divided into four groups and cultured in osteogenic differentiation media. MSCs treated with 25 μM SB203580 (co-M+SB203580), 10 μM FR180204 (co-M+FR180204), or 10 μM SP600125 (co-M+SP600125). Inhibitor free group was used as control (co-M).

### Microarray hybridization and data analysis

Three wells of co-MSCs were prepared for microarray analysis and MSCs alone were used as the control. The osteogenic differentiation will spend about 14 days, total RNA was extracted using TRIzol® Reagent according to the manufacturer’s instructions (Invitrogen, USA), and genomic DNA was removed using DNase I (TaKara, Japan). An RNA-seq transcriptome library was prepared following instructions from the TruSeqTM RNA sample preparation Kit (Illumina, USA). Libraries were size selected for cDNA target fragments of 200–300 base pairs (bp) on 2% low range ultra agarose, followed by PCR amplification using Phusion DNA polymerase (NEB, USA) for 15 PCR cycles. After quantification by TBS380, the paired-end RNA-seq library was sequenced on the Illumina Novaseq 6000 (2 × 150 bp read length). The expression level of each transcript was calculated according to the FPKM method. RSEM (http://deweylab.biostat.wisc.edu/rsem/) was used to quantify gene abundance. Volcano plot was performed by R statistical package software, and EdgeR was used for differential expression analysis [25]. Gene ontology (GO) functional enrichment and KEGG pathway analyses were conducted on GOATOOLS (https://github.com/tanghaibao/Goatools) and KOBAS (http://kobas.cbi.pku.edu.cn/download.php) [26]. Microarray data were analyzed on the online platform, Majorbio Cloud Platform (www.majorbio.com).

### Quantitative reverse transcription-polymerase chain reaction (qRT-PCR)

Total RNA was isolated at 14 days after incubation. RNA quality was determined on 2100 Bioanalyzer (Agilent, USA) and quantified using the Nanodrop, ND-2000 (NanoDrop Technologies, USA). RNA reverse transcription was performed using the SuperScript™ kit (Invitrogen, USA) and synthesized cDNA was used to perform qRT-PCR reactions [27]. PCR amplifications were performed using specific primers (Table 1) for analyzing the expression of osteopontin (OPN), bone sialoprotein (BSP), Runt-related transcription factor 2(Runx2), TGF-beta activated kinase binding protein 1 (TAB1), MKK6, and p38. Real time-PCR conditions were 95 °C for 1 min, followed by 95 °C for 30 s, and then 58 °C for 40 s, over 35 cycles [24]. The experiments are performed at least three times. GAPDH served as a housekeeping gene. The Ct-method (2^-ΔΔCT^) was adopted for gene expression calculations.

**Table 1:** Primers sequences for qRT-PCR

Gene	Forward primer (5′-3′)	Reverse primer (5′-3′)
OPN	GAGGAGGCAGAGCACAGCAT	GCAAAAGCAAATCACTGCAATT
BSP	CTGTAGCACCATTCCACACT	ATGGCCTGTGCTTTCTCGAT
Runx2	CCCGTGGCCTTCAAGGT	CGTTACCCGCCATGACAGTA
TAB1	TGGAAAGATCAAGCAGGTGG	GATTGGTTTGGACTTGGCAG
MKK6	CCAGACAATTCCAGAGGACATC	CACATCTTCACTTGACCGAGAG
P38	TGAAATGACAGGCTACGTGG	CTTCCAGTCAACAGCTCGG
GAPDH	TGTGTCCGTCGTGGATCTGA	TTGCTGTTGAAGTCGCAGGAG

### Western blotting

Proteins were processed with the whole cell lysis assay kit (Keygen, China). Equal protein concentrations from each cell lysate were subjected to 10% SDS-PAGE and then transferred to polyvinylidene fluoride (PVDF) membranes (Millipore, USA). Membranes were blocked in 5% bovine serum albumin at room temperture and probed with the following antibodies at 4 °C overnight: OPN, BSP, RUNX2, Col I, JNK, phosphorylated-JNK, p38, phosphorylated-p38, ERK1/2, and phosphorylated-ERK1/2(Abcam, USA) at a dilution range of 1:500–1:1000. After this, membranes were washed 3 times with TBS-T and incubated with horseradish peroxide-conjugated secondary antibodies for 60 min at room temperature. Enhanced chemiluminescence reagents (Millipore, USA) were used to observe immunoreactive proteins, blots were quantified using Image J software, and measurements were performed in triplicate.

### ALP activity assay

MSCs were harvested at 7, 14, and 21 days. ALP activity was determined using an ALP assay kit (Sigma, USA) according to the manufacturer’s instructions. A BCA protein assay kit (Beyotime, China) was used to calculate total protein content for ALP activity normalization, and measurements were performed in triplicate.

### Calcium mineral deposition

Calcium deposits were determined by the Osteogenesis Quantitation Kit (Sigma-Aldrich, USA). Briefly, cells were washed with PBS twice. Then cells were fixed with 10% formaldehyde and incubating at room temperature for 15 min. Subsequently, cells were washed with distilled water for 3 times and stained with 1x Alizarin Red at room temperature for 20 min. After acid extraction, extracted solution was measured at 405 nm. A serial dilution of ARS standards was prepared for quantitative analysis according to the manufacturer’s instructions, and measurements were performed in triplicate.

### p38 siRNA interference

For p38 silencing, siRNA against p38 (JTS, China) was transfected into MSCs using Lipofectamine 3000 transfection reagent (Thermo Fisher, USA) according to manufacturer’s instructions. A siRNA negative control sequence was used as a control. Cells were incubated at 37 °C for 48 h, and then treated with osteogenic inducing media. Monolayer MSCs and MSCs/EPCs co-culture groups treated with p38 siRNA or control siRNA were defined as (1) MSCs+ control siRNA group (M + NC), (2) MSCs+p38 siRNA group (M + si), (3) MSCs/EPCs co-culture + control siRNA group (co-M + NC), and (4) MSCs/EPCs+p38 siRNA group (co-M + si). Alkaline phosphatase activity assay and Alizarin Red staining were performed on cells. Western blotting was performed to detect OPN, BSP, and RUNX2 protein levels, and blots were quantified using Image J software in triplicate.

### Statistical analyses

Statistical analysis was performed using SPSS-17.0 software. All data are expressed as the mean ± SD. Statistical analyses were performed with one-way analysis of variance or Student’s *t* test. The probability level at which differences were considered statistically significant was *P* < 0.05.

## Results

### Isolation and characterization of MSCs and EPCs

**Fig. 1:**
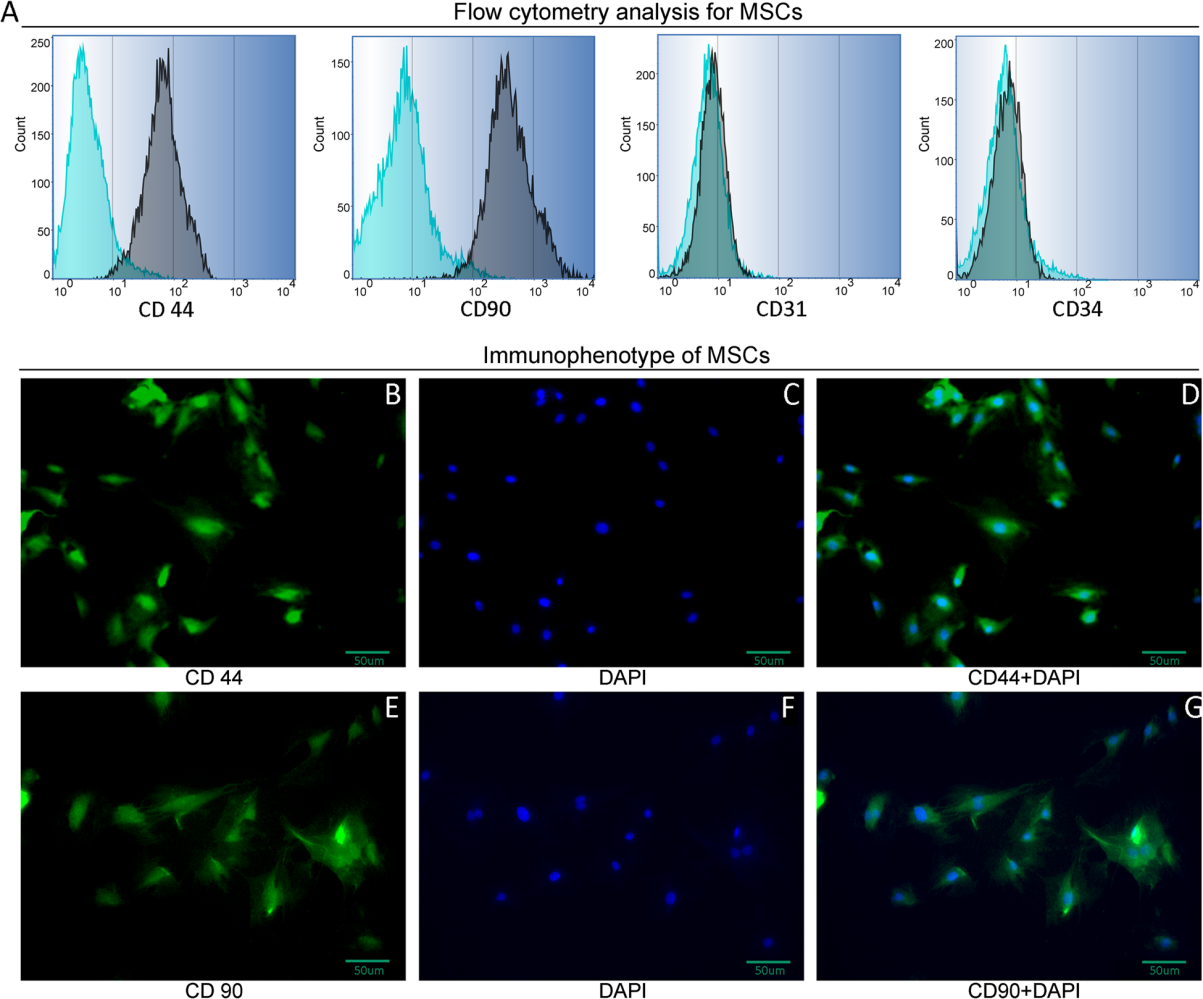
Phenotype identification of MSCs. **a** Flow cytometry analysis for MSCs. Cells were stained positive for MSCs surface markers CD44 and CD90 and negative for CD31 and CD34. **b** FITC labeled CD44 fluorescence staining of MSCs cultured for 10 days (scale bar = 50 μm). **c** DAPI nuclear staining (blue). **d** Overlay of CD44 and DAPI staining. **e** FITC labeled CD90 fluorescence staining of MSCs. **f** DAPI nuclear staining (blue). **g** Overlay of CD90 and DAPI staining

**Fig. 2:**
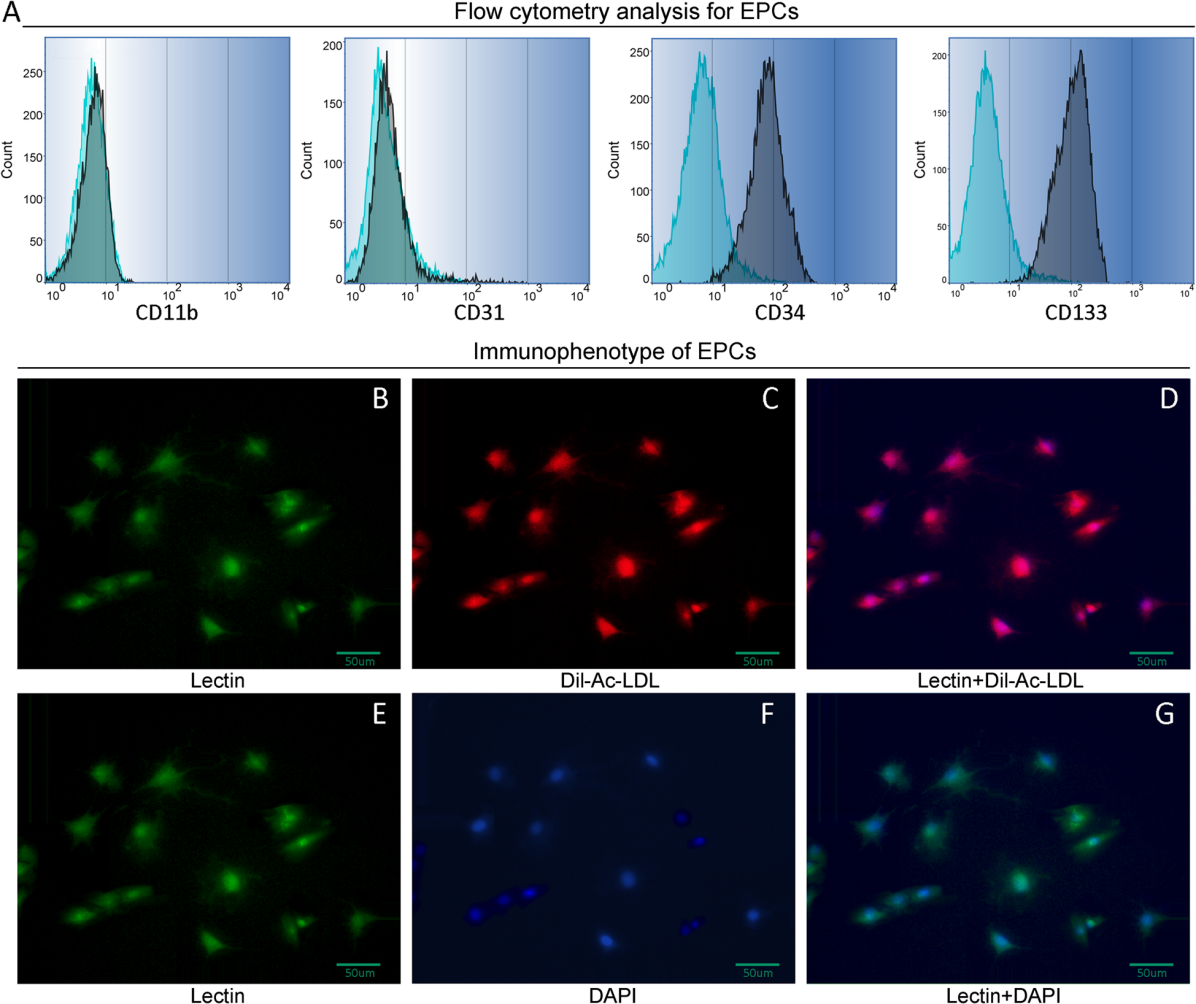
Phenotype identification of EPCs. **a** flow cytometry analysis for EPCs. Cells were positive for CD34 and CD133 and negative for CD11b and CD31. **b** Lectin fluorescence staining for EPCs cultured for 10 days. Scale bar = 50 μm. **c** Uptake of Dil-Ac-LDL staining for EPCs (red). **d** Overlay of Lectin and Dil staining. **e** Lectin fluorescence staining (green). **f** DAPI nuclear staining (blue). **g** Overlay of lectin and DAPI staining

Cells were analyzed and identified by flow cytometry analysis and immunofluorescence staining for MSCs cell surface markers at passage four. As shown in Fig. 1a, MSCs expressed cell-surface protein profiles, positive for CD44 and CD90 and negative for CD34 and CD31, and we also observed the expression of CD44 and CD90 by immunofluorescence staining (Fig. 1b–g) and the results showed that the cells were positively stained with FITC-labeled CD44 and CD90. To confirm EPC phenotype, we analyzed the hematopoietic progenitor cell marker by flow cytometry. Results showed that EPCs were positive for FITC-labeled CD34 and CD133 and negative for CD11b and CD31 (Fig. 2a). We observed expression of lectin and Dil-ac-LDL by immunostaining. As shown in Fig. 2b–g, cells were stained positive for FITC labeled lectin (green) and took up Dil-ac-LDL (red).

### Differentially expressed genes in co-cultured MSCs

**Fig. 3:**
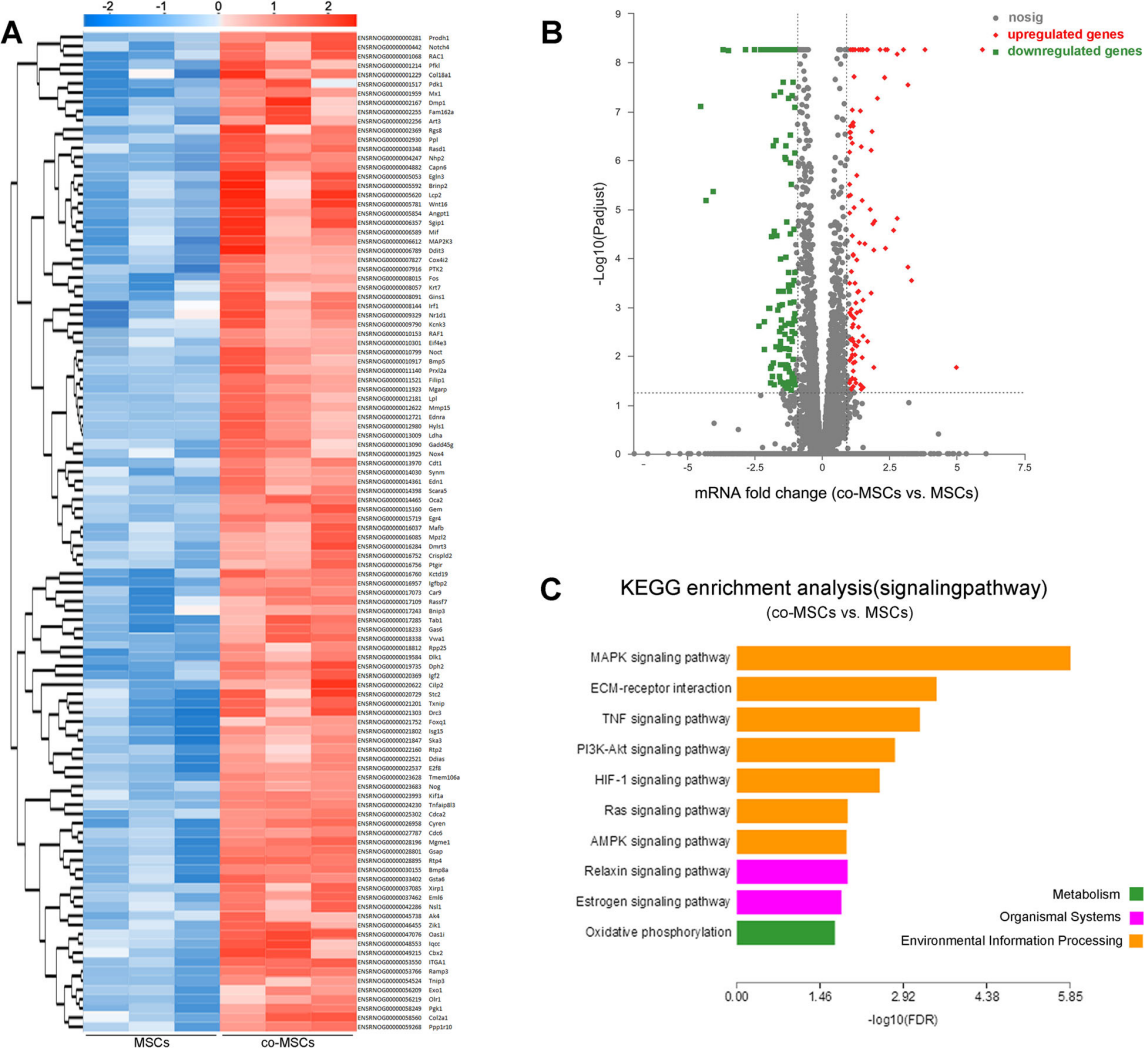
PCR array analysis of co-cultured MSCs, monolayer culture MSCs were used as control. **a** Heat map displaying hierarchical clustering of differentially expressed genes from co-cultured MSCs. Upregulated genes are shown in red, whereas downregulated genes are shown in green. **b** Volcano plot of microarray data. The horizontal bar represents the nominal significant level of 0.01 for Student’s *t* tests, the vertical dashed bars denote ≤ 0.6-fold downregulation (left) or ≥ 0.6-fold upregulation (right). **c** KEGG enrichment analyses of targeted genes indicated that the effects of EPCs on the regulation of MSCs are associated with MAPK signaling pathway, ECM-receptor interaction, TNF signaling pathway, PI3K-Akt signaling pathway, HIF-1 and Ras signaling pathway, and so on

In view of accumulating evidence suggesting that EPCs increase osteogenic gene expression during osteogenic induction of co-cultured MSCs. We used a cDNA microarray technique to identify the differentially-regulated genes in co-MSCs versus monolayer cultured MSCs during osteogenic induction. Heat map displayed hierarchical clustering of differentially expressed genes from co-cultured MSCs. Upregulated genes are shown in red, whereas downregulated genes are shown in green (Fig. 3a). Volcano plot revealed that out of 237 differentially expressed genes, 109 were upregulated and 128 genes were downregulated in co-MSCs (Fig. 3b). KEGG enrichment analyses of differentially expressed genes demonstrated that many signaling pathways, including MAPK, ECM-receptor interaction, TNF, PI3K-Akt, and HIF-1, were regulated by co-cultured EPCs (Fig. 3c). Among these pathways, MAPK was primarily affected by co-cultured with EPCs. These data provided basic data for further investigation.

### EPCs increase osteogenic gene expression of indirectly co-cultured MSCs

**Fig. 4:**
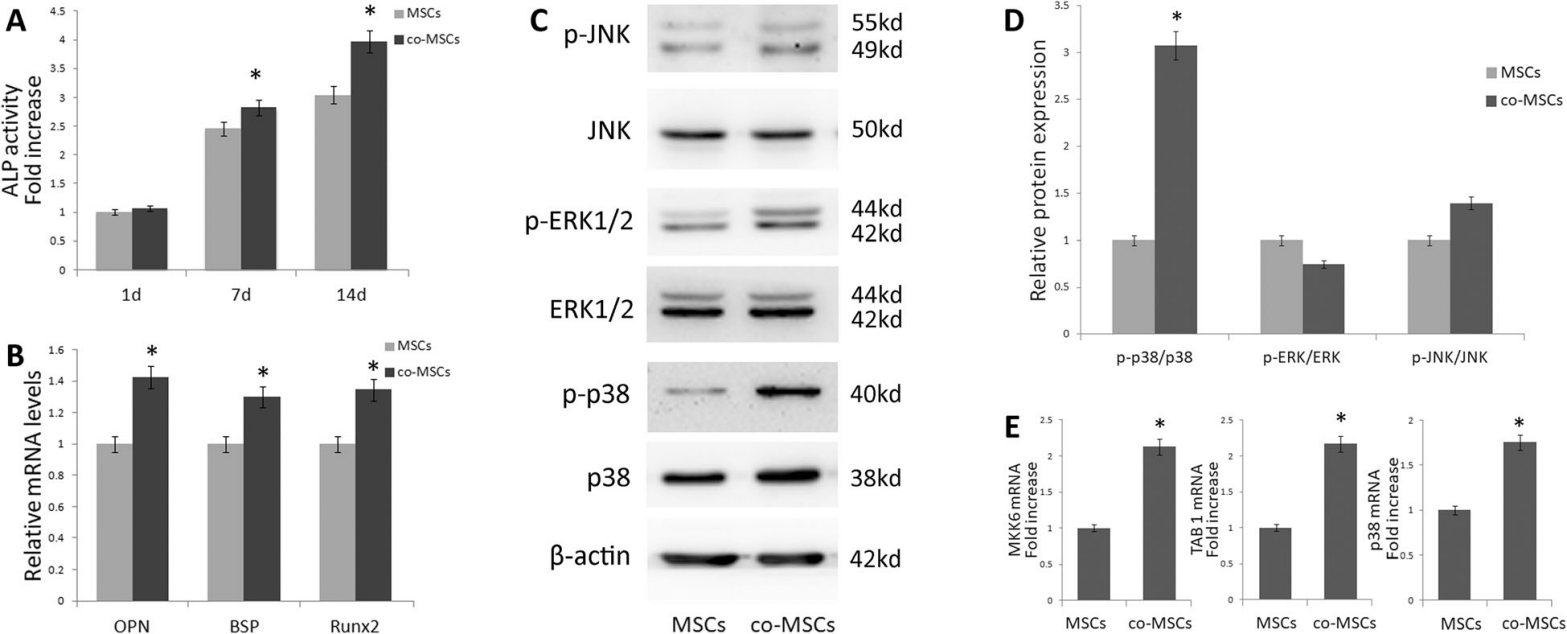
EPCs promotes the osteogenic differentiation of MSCs. **a** Osteogenic differentiation was determined by ALP staining at day 7, 14, and 21 days after osteogenic differentiation induced. Data represents the mean ± SD of *n* = 3 samples. **b** Expression of the osteogenic genes OPN, BSP, and Runx2 were measured by qRT-PCR at the day 14 after osteogenic differentiation induced (*n* = 3) *p < 0.05. **c** EPCs promotes activation of the MAPK signaling pathway of MSCs. p38, p-p38, ERK1/2, p-ERK1/2, JNK, and p-JNK expression were examined by western blotting, *p < 0.05. **d** Densitometry of protein levels of p38, p-p38, ERK1/2, p-ERK1/2, JNK, and p-JNK by immunoblotting, β-actin was used as the internal control. Data are presented as the mean ± SD. And **e** MKK6, TAB1, and p38 mRNA expression were measured by qRT-PCR (*n* = 3), *p < 0.05. Co-MSCs, co-cultured MSCs; MSCs, monolayer cultured MSCs

To examine the effect of EPCs on the differentiation of MSCs, MSCs were cultured in osteogenic induction media. The study showed that MSCs behave differently in terms of differentiation when cultured in association with EPCs. An increase in ALP was observed when MSCs were co-cultured with EPCs. ALP activity was measured at days 1, 7, and 14 and quantified by ALP assay kit. Our data showed that when MSCs were co-cultured with EPCs, an increased ALP activity was observed and the highest ALP activity was found at 14 days, when compared with day 1 and 7; EPCs significantly increased ALP activity of MSCs at 7 (*P* < 0.05) and 14 (*P* < 0.01) days, by 14.91% and 30.52% (Fig. 4a).

qRT-PCR at day 14 was performed to quantitative osteogenic gene expression, including Runx2, OPN, and BSP, to investigate the effects of EPCs on MSCs differentiation. Results showed that EPCs increased OPN mRNA expression significantly by 42.16% in co-cultured MSCs (*P* < 0.05), and BSP and Runx2 mRNA levels also increased significantly in co-cultured MSCs by 29.87% and 34.52%, respectively, in comparison with monolayer MSCs (Fig. 4b).

### EPCs promote TAB1, MKK6 and p38 gene expression, and p38 phosphorylation

qRT-PCR and western blotting were used to assess whether MAPK pathways were involved in osteogenesis. When compared with monolayer cultured MSCs, p38 phosphorylation levels were significantly upregulated in EPCs co-cultured MSCs. However, JNK and ERK1/2 phosphorylation levels were not significantly altered (Fig. 4c, d). qRT-PCR data showed that p38 gene expression was significantly upregulated by 75.27% (*P* < 0.05) in the MSCs/EPCs co-culture group. TAB1 and MKK6 gene expression was also significantly upregulated by 116.93 and 112.51% (*P* < 0.05) in the MSCs/EPCs co-culture group (Fig. 4e).

### EPCs promote osteogenic differentiation through promoting p38 MAPK signaling pathway activation

**Fig. 5:**
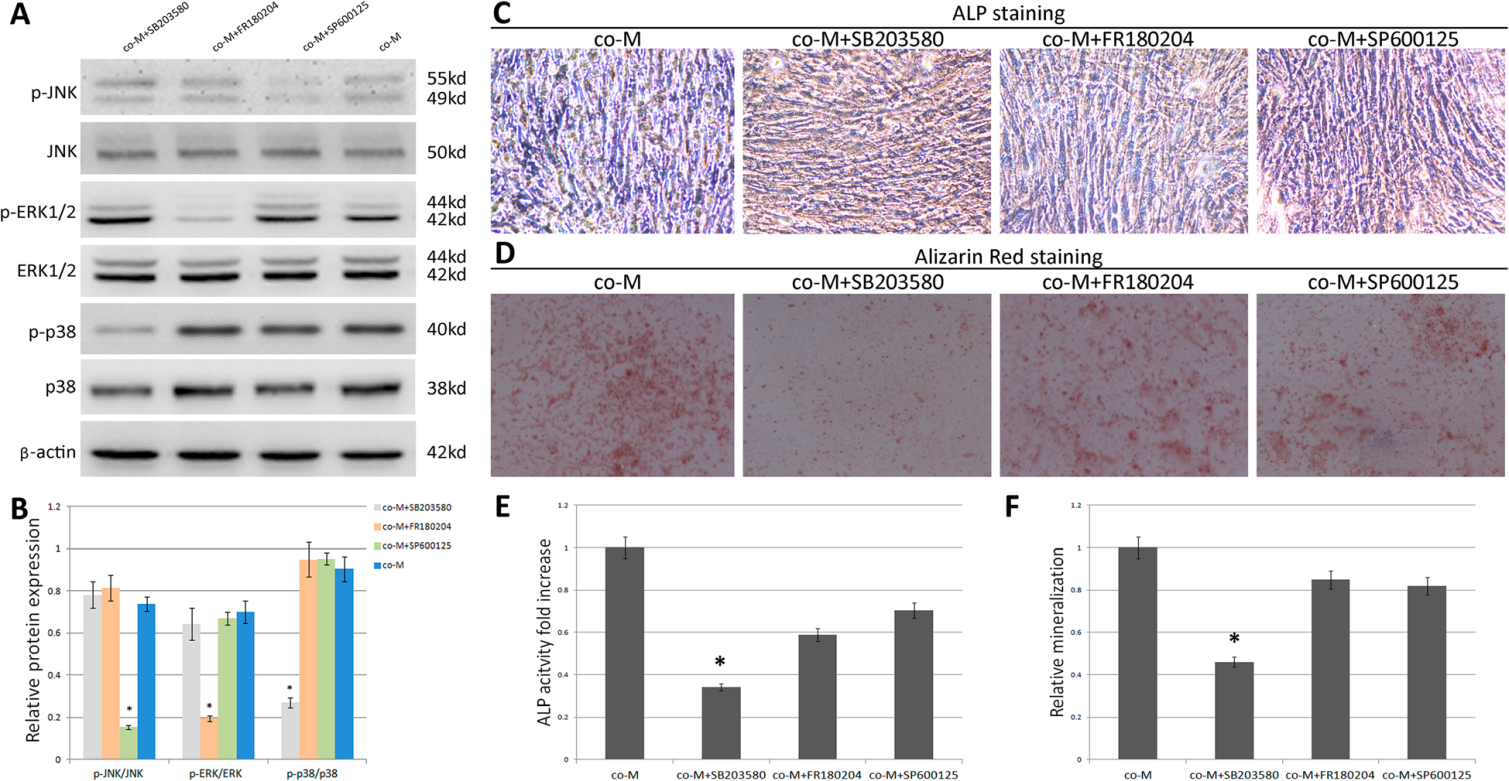
EPCs induce osteogenic differentiation by promoting MAPK phosphorylation. MSCs were pretreated with p38 signaling inhibitor SB203580, ERK signaling inhibitor FR 180204, or JNK signaling inhibitor SP600125 and then co-cultured with EPCs and examined for **a** the protein levels of p-p38, p38, p-ERK1/2, ERK1/2, p-JNK, and JNK by immunoblotting. **b** Scanning densitometry of relative protein levels of p-p38, p38, p-ERK1/2, ERK1/2, p-JNK, and JNK by immunoblotting, β-actin was used as the internal control. Data are presented as the mean ± SD, *p < 0.05. **c** Osteogenic differentiation was determined by ALP staining. **d** Calcium nodules formation was determined by Alizarin Red staining. **e** Relative ALP activity (*n* = 3), *p < 0.05. **f** Relative mineralization levels (*n* = 3), *p < 0.05

To further investigate whether EPCs promote the osteogenic differentiation of MSCs via MAPK signaling pathway, we used p38 signaling inhibitor SB 203580, ERK signaling inhibitor FR180204, and JNK inhibitor SP600125 to evaluate the changes in MAPK signaling and osteogenic differentiation in co-cultured MSCs. Following treatment with the inhibitors of specific pathway, the protein and their phosphorylated protein levels of p38, ERK1/2, JNK, and osteogenic capacities of MSCs were determined. Results showed that p38, ERK1/2, and JNK phosphorylation levels reduced significantly via SB203580, FR180204, and SP600125 (Fig. 5a, b). This regulation of the osteogenic differentiation capacity of MSCs via MAPK signaling pathway was further confirmed by ALP assay and alizarin red staining. Results showed that the ALP levels and calcium nodules formation were reduced significantly by SB203580 (Fig. 5c–f). The results demonstrated that p38 MAPK is involved in osteogenic differentiation of MSCs.

### The effects of p38 siRNA on the osteogenic differentiation of MSCs

**Fig. 6:**
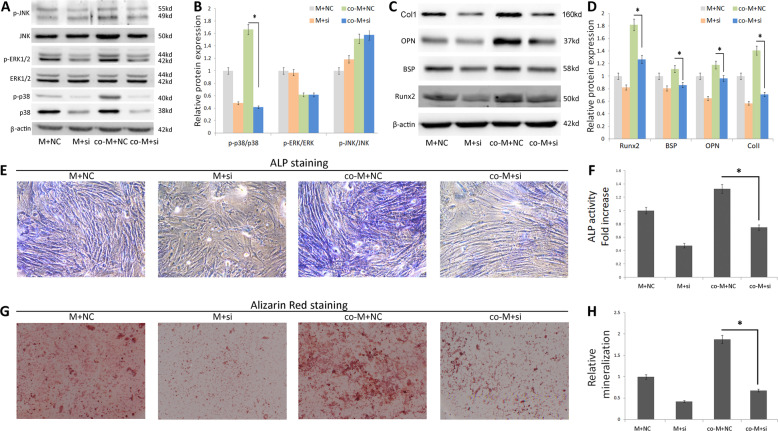
p38 MAPK signaling is involved in MSCs osteogenic differentiation. MSCs were transfected with siRNA-p38 and examined for **a** the protein expression of p-p38, p38, p-ERK1/2, ERK1/2, p-JNK, and JNK by immunoblotting, β-actin was used as the internal control. **b** Densitometry of relative protein levels of p-p38, p38, p-ERK1/2, ERK1/2, p-JNK, and JNK, (*n* = 3), *p < 0.05. **c** The protein expression of OPN, BSP, Runx2, and Col I by immunoblotting. **d** Densitometry of relative protein levels of OPN, BSP, Runx2, and Col I, (*n* = 3), *p < 0.05. **e** Osteogenic differentiation was determined by ALP staining. **f** Relative ALP activity (*n* = 3), *p < 0.05. **g** Calcium nodules formation by Alizarin Red staining. **h** Relative mineralization levels (*n* = 3), *p < 0.05

We demonstrated that the p38 signaling pathway plays an important role in co-cultured MSCs. After this observation, we used p38 siRNA silencing to further explore the function of p38MAPK in promoting osteogenic differentiation in MSCs. p38 siRNA was used to prevent p38MAPK pathway activation. We observed that p38 phosphorylation levels were significantly decreased after p38 siRNA supplementation to cells. However, p-JNK and p-ERK1/2 expression levels were not significantly altered (Fig. 6a, b). Next, we examined the effects of p38 siRNA on the osteogenic differentiation of MSCs. As shown in Fig. 6, p38 silencing significantly reduced Runx2, OPN, BSP, and Col I protein expression levels (Fig. 6c, d). Consistently, ALP levels and calcium nodule formation were both suppressed by p38 silencing (Fig. 6e–h). Therefore, EPCs have positive effects on osteogenic differentiation of MSCs through activating p38 MAPK signaling pathway.

## Discussion

Bone tissue engineering based on stem cells and biomaterials provides a strategy to reconstruct the impaired bone. Among cells with therapeutic potential, MSCs have gained much interest. MSCs can self-renewal and differentiate into multiple cell types, they are regarded as suitable cell sources for bone tissue engineering [28]. The ability of MSCs to differentiate into multiple lineages is controlled by various inhibitors and stimulators, which have been studied by in vivo or in vitro [29, 30]. Enhanced MSCs osteogenic differentiation can be achieved by various methods, such as electrical stimulation, chemical supplements, and co-culturing with assisting cells [31, 32]. Co-culture is a strategy to culture multiple cell types, directly or indirectly, in the same microenvironment. Taking the advantage of this technique, co-culture has the ability to control behavior and actions of target cells through feedback or crosstalk mechanisms.

The use of MSCs/EPCs co-culture is an effective method to promote osteogenic differentiation. It has been reported that co-seeding of EPCs and MSCs will result in the efficient formation of bone [33]. Our previous study also demonstrated that EPCs enhanced MSC osteogenesis in vivo [24]. In line with our observation, EPCs can promote significant osteogenic differentiation of MSCs by co-culturing techniques [16, 34]. In the case of bone tissue engineering, interactions between endothelial progenitors and stem cells are currently the focus of interest [35–37], and an understanding of cellular and molecular interactions between endothelial progenitors and stem cells will enhance the development of bone regeneration. Therefore, we co-cultured MSCs with EPCs in Transwell chambers indirectly to determine the effects of EPCs on influencing the osteogenic differentiation of MSCs. Although the indirect co-culture system could not completely mimic the spatial relationship between the two types of cells, it avoided the difficulty of cell separation in a direct co-culture system [38]. Previous studies have confirmed that intercellular contact signaling is a key determinant for MSCs fate [23]. Endothelial differentiation of MSCs only occurs in direct co-culture systems. Direct contact with EPCs can induce endothelial phenotypes, and angiogenesis in MSCs, or differentiate MSCs into pericyte-like cells [39, 40], whereas MSCs maintain their initial biological properties in in-direct culture conditions [41, 42]. Co-culturing systems control cell behaviors and actions, and like many researchers, we used osteogenic medium to analyze the differentiation of MSCs [34, 43]. It was proved that osteogenic inducing media might not induce osteogenic differentiation for EPCs [44], which suggest that EPCs maintained their initial biological properties when co-cultured with MSCs. Cellular interactions are key factors in co-culture microenvironments. Studies have demonstrated that factors generated by EPCs are considered crucial for the process of osteogenesis, and suggest that paracrine signaling was sufficient to promote MSCs differentiation [16].

Our data demonstrated that co-cultured MSCs exhibited higher ALP activities than monolayer cultured MSCs, and EPCs promoted the co-cultured MSCs osteogenic mRNA expression of OPN, BSP, and Runx2, by 42.16%, 29.87%, and 34.52%, respectively, in comparison with MSCs monolayer culture. The results indicate that EPCs had a profound effect on differentiation and osteogenesis of MSCs in co-culture.

Osteogenic differentiation processes involve transcription factors, growth factors, and signaling pathways, which transmit molecular information to allow cells react to external stimuli [45]. To determine candidate signaling pathways potentially involved in osteogenesis, we performed microarray analysis to identify transcriptional profiles and showing the differential expression of genes in co-cultured MSCs. Results revealed that out of 237 differentially expressed genes, 109 were upregulated in co-cultured MSCs and 128 genes were downregulated. KEGG enrichment analyses of targeted genes indicated that the effects of EPCs on the regulation of MSCs biological properties are most associated with MAPK signaling pathway, ECM-receptor interaction, PI3K-Akt signaling pathway, Ras and HIF-1 signaling pathway, and so on. Among these pathways, MAPK signaling pathway was primarily affected by co-cultured with EPCs.

MAPKs play important roles in controlling cell proliferation and are involved in multiple cellular pathways and functions [46, 47]. MAPK functions are mediated via the phosphorylation of several substrates, including phospholipases, transcription factors, and cytoskeletal proteins [48]. Studies have demonstrated the effects of ERK, p38, and JNK MAPKs on bone formation. Among these signaling pathways, ERK1/2 are kinase signals that are activated by growth factors, while p38 were activated by stress and cytokines [48].

In this study, we observed that the MAPK signaling pathway was involved in MSCs osteogenesis. Our qRT-PCR data showed that MKK6, which initiates the MKKs-p38MAPK or -ERK signaling cascade [49], increased significantly in co-cultured MSCs. In DNA array, we found that TAB1, instead of TAK1, was significantly upregulated. This observation was further corroborated by qRT-PCR. TAB-1 was found to interact with TAK-1, which is identified as an alternative pathway, leading to the activation of p38. It is also suggested that TAB-1 can directly bind to p38, which results in promoting autophosphorylation of p38 [50]. We detected osteogenesis-related p38MAPK, ERK1/2, and JNK pathway proteins by western blot analysis and observed the increased phosphorylation of the p38MAPK pathway, but no significant changes in ERK1/2 and JNK signaling. Moreover, the p38 inhibitor SB203580 suppresses EPCs enhanced mineralization and ALP activity in co-cultured MSCs, while ERK1/2 and JNK inhibitors have no effect. Interestingly, p38 mRNA expression in co-MSCs increased significantly, but total protein of p38 did not increase according to mRNA expression. This may be due to the presence of post-transcriptional regulation, such as the regulation of miRNA. EPCs promoted p38 phosphorylation rather than increase p38 total protein in MSCs. The results suggest that the p38 MAPK signaling pathways may be involved in EPCs increased osteogenic differentiation of MSCs.

p38MAPK signaling pathway is involved in osteoblast proliferation and differentiation [51, 52]. Bone mineral density significantly decreased in osteoblast-specific p38 knockout mice [53]. To further investigate whether EPCs promote the osteogenic differentiation of MSCs via MAPK signaling, we confirmed the results by siRNA knockdown. Results showed that p38 siRNA significantly decreased p38 phosphorylation levels and significantly reduced Runx2, OPN, BSP, and Col I protein production. ALP levels and calcium nodule formation were also both suppressed in the p38 silencing group. Consistent with previous studies, it was reported that p38MAPK participates in ALP regulation during osteoblast differentiation, and is necessary for BSP and OPN expression, via p38MAPK-Runx2 activation [54, 55], lack of p38α in pre-osteoblasts results in defective osteoblast differentiation, as evidenced by reduced expressions of collagen 1, ALP, BSP, and OCN [56]. The administration of selective p38 inhibitors on osteoblasts showed that p38 modulates osteogenic differentiation, extracellular matrix deposition, and mineralization. p38MAPK also promotes hBMSCs osteogenic differentiation via regulating Runx2/Osx [57]. Reports have also indicated that MSC-derived exosomes promoted the proliferation of osteoblast via MAPK pathway [58]. Based on these studies, we speculate EPCs enhance osteogenic differentiation via the p38MAPK signaling pathway in co-cultured MSCs.

This observed effect may be exerted through exosomes or a variety of paracrine mechanisms [59, 60]. It was proved that EPCs create local nutritional support environments by producing microvesicles, exosomes, and growth factors which promote cell proliferation and cell biological motility maintenance [61–63]. It was reported that EPCs derived exosomes transplantation significantly accelerate bone regeneration in rats [37]. Moreover, EPCs derived exosomes could significantly promote MSCs proliferation, differentiation, and the expression of osteogenic genes [64]. MSCs endocytosed exosomes via the caveolar will trigger the activation of p38MAPK pathway by phosphorylating mechanism [65]. Researchers also found that EPCs enhance osteogenesis by stimulating proliferation of surrounding cells via a paracrine effect [66], such as VEGF and its receptor, which reported to induce the osteogenic differentiation of stem cells, recruit major bone-forming cells, and stimulating the release of BMP-2 by osteoblast [67–69]. Cellular interactions that take place between MSCs and EPCs benefit both cell types [70]. These interactions are complex and seldom unidirectional, which means that both cell types were affected in the co-culture system. A combination of cellular and molecular factors is necessary for the co-culture cell rearrangement. These factors could be dependent upon the intercellular crosstalk between MSCs and EPCs.

All these results suggested that important cellular interactions occur between EPCs and MSCs. Further studies are required to explore the molecular mechanisms that mediate MSCs differentiation and to elucidate the roles of EPCs in the processes. The findings of the present study may promote the understanding of tissue repair mechanisms and may lead to the development of bone tissue engineering.

## Conclusion

The results of this study support the concept that EPCs can enhance MSCs osteogenic differentiation in vitro*.* EPCs have profound effects on differentiation and upregulates osteogenesis of MSCs. EPCs significantly promoted MSCs osteogenic gene expression and protein synthesis. ALP activity and calcium nodule formation were also increased significantly. Exosomes or paracrine factors from EPCs influence the biological function of MSCs; activation of the p38MAPK pathway may be the key to enhancing MSCs osteogenic differentiation. Further insights into the cellular interactions will be invaluable for understanding in vitro cell dynamics, ultimately leading to more rational tissue engineering strategies.

## Data Availability

The datasets used or analyzed during the current study are available from the corresponding author on reasonable request.

## Abbreviations

EPCs: Endothelial progenitor cells
MSCs: Mesenchymal stem cells
ALP: Alkaline phosphatase
MAPK: The mitogen-activated protein kinase
PBS: Phosphate buffered saline
OPN: Osteopontin
BSP: Bone sialoprotein
Runx2: Runt-related transcription factor 2
TAB1: TGF-beta activated kinase binding protein 1
